# Economic Well-being and Associated Mediating Pathways to Improved Antiretroviral Therapy Adherence Among Adolescents Living With HIV: A Prospective Cohort Study in South Africa

**DOI:** 10.1097/QAI.0000000000003071

**Published:** 2022-08-15

**Authors:** Janina Isabel Steinert, Yulia Shenderovich, Michael Smith, Siyanai Zhou, Elona Toska, Lucie Cluver

**Affiliations:** a TUM School of Social Sciences and Technology, Technical University of Munich, Munich, Germany;; b Centre for Evidence-Based Intervention, Department of Social Policy and Intervention, University of Oxford, United Kingdom;; c Wolfson Centre for Young People's Mental Health, Cardiff University, Cardiff, United Kingdom;; d Centre for the Development and Evaluation of Complex Interventions for Public Health Improvement (DECIPHer), School of Social Sciences, Cardiff University, Cardiff, United Kingdom;; e United Nations World Food Programme, Nutrition Division, Rome, Italy;; f Centre for Social Science Research;; g Division of Social and Behavioural Sciences, School of Public Health and Family Medicine; and; h Department of Sociology, University of Cape Town, Cape Town, South Africa.

**Keywords:** antiretroviral therapy adherence, poverty, adolescents, South Africa, cohort study

## Abstract

Adolescents living with HIV exhibit lower levels of adherence to antiretroviral therapy (ART) than other age groups. Poverty is a key barrier to ART adherence. This study aims at understanding how alleviating poverty through structural and internal pathways can help increase ART adherence among adolescents.

**Setting:**

Eastern Cape province, South Africa.

**Methods:**

One thousand forty-six adolescents living with HIV were recruited from 53 public health care facilities and interviewed at 3 data collection waves with a retention rate of 89% and a mortality rate of 3%. Data were collected by face-to-face, device-assisted interviews. Hybrid probit regressions and a structural equation path analysis were used to estimate the association between poverty reduction (increased access to basic necessities) and the pathways by which it could improve ART adherence.

**Results:**

Self-reported ART adherence ranged from an average of 66% (n = 615) at baseline to 75% (n = 700) in the last wave. Within-person and between-person improvements in economic well-being were associated with significant increases in adolescent ART adherence. On average, adolescents with access to 3 additional basic needs experienced a 4 percentage-point increase in the probability of ART adherence. Structural pathways to improved ART adherence included participants having enough money to travel to the clinic and sufficient food to eat when taking medication. Internal pathways included improved psychological well-being and reduced internalized HIV stigma.

**Conclusion:**

Poverty reduction programs such as HIV-sensitive social protection can address structural and psychological pathways to increase ART adherence among economically disadvantaged adolescents by incentivizing demand-side interventions and the provision of quality essential services.

## Abstract 2

Supplemental Digital Content is Available in the Text.

## INTRODUCTION

Globally, 2 million adolescents aged 10–19 years are living with HIV.^1^ Almost 20% of these live in South Africa alone, the country with the highest number of HIV infections worldwide.^1,2^ Adolescents living with HIV (ALHIV) in South Africa do not yet access antiretroviral therapy (ART) at the desired rate of the UNAIDS 90-90-90 treatment target.^3^ Even if on ART, adolescents show lower adherence rates compared with all other age groups.^2,4^ Estimates suggest that along the care cascade only 62% of South African adolescents know their HIV status, 65% of whom are on treatment, and only 78% of those on treatment are fully virally suppressed.^5^ Poor adherence is associated with higher morbidity and mortality rates, drug resistance, and higher onward transmission risk.^6^ HIV/AIDS—despite the availability of life-saving treatment and a national universal test-and-treat approach—thus remains the leading cause of death among adolescents and young people in South Africa.^7^ Adolescents with inconsistent ART adherence exhibited higher rates of mental health problems such as depression, anxiety, and suicidality.^8–10^ A nuanced understanding of adherence barriers among adolescents—and strategies to alleviate these—is thus paramount.

A review published in 2015 has identified economic deprivation as one of the key risk factors of low levels of ART adherence among adolescents in Sub-Saharan Africa.^4^ Likewise, empirical analyses have repeatedly pointed to poverty and lack of financial resources as central barriers for retention in HIV care, particularly in low-income and middle-income countries.^6,11–13^ While experimental and quasi-experimental studies suggest that *reducing* poverty through economic empowerment interventions is causally related to improvements in ART adherence,^14–16^ longitudinal observational evidence on how poverty alleviation can improve ART adherence is still scarce and only few studies to date have specifically focused on adolescents.^12,17–19^ For example, a longitudinal study with 637 ALHIV in Uganda identified poverty as one of 5 key predictors of poor ART adherence, out of a list of 17 potential predictors.^17^ A qualitative study conducted with adolescents from low socioeconomic urban neighborhoods in Cape Town identified the lack of financial support, for example, to cover fees for public transport to clinics, as a key barrier to ART adherence.^19^ Finally, a mixed-methods study with 17–19-year-old ALHIV in Zambia found that inadequate nutrition and having to walk to the clinic appointment were inhibiting factors for taking ARVs.^18^

Poverty might affect ART adherence through various channels. First, there are *structural pathways* linked to poverty and economic hardship that may determine access to ART. For example, several studies have found that the costs for transportation to and distance from health clinics can be a major burden to low-income patients living with HIV, particularly for those living in more remote and rural areas, where accessible transport is limited or not available.^11,14,20–22^ Specifically, a meta-analysis of patient-reported barriers to ART adherence revealed travel costs as the second most important obstacle to accessing care, mentioned by 40% of ALHIV.^23^ Another study was conducted in Kwazulu-Natal, South Africa, with 500 patients living with HIV, of which 300 were on ART and 200 were classified as “pre-ART.” The study found that patients on ART had significantly higher expenditures (+ZAR 34.0) on transport to clinics relative to the pre-ART patients. Relatedly, 39% of ART patients, compared with 31% of pre-ART patients, indicated that they had accumulated debt or sold assets to cope with the increased health care costs.^24^ Apart from travel costs, geographic remoteness can be a key determinant of ART adherence. For example, a study conducted with 3695 serodiscordant couples in the Henan province of China found that for those living within medium distance (ie, 5–10 km) from their assigned HIV clinic, compared with those living closer to the clinic (<5 km), HIV transmission risk was significantly higher.^25^ Another analysis based on 26,365 clinical records from patients in PEPFAR-supported facilities in Nigeria found that adolescents and children in rural regions had a significantly higher risk of nonadherence.^26^ In addition, ART adherence may be hindered through food insecurity and, thus, the inability to eat before taking one's ARVs.^11,12,27,28^ This can be particularly challenging for growing adolescents and those at early treatment stages who may experience increased appetite as a result of initiating treatment.^18,20^

In addition, poverty may reduce adherence to ART through individual-level *internal pathways*. An emerging body of literature has documented experimental evidence on the links between economic scarcity and mental illness, including elevated levels of stress, depression, anxiety, and poor cognition induced by negative income shocks.^29–32^ Poor mental health, in turn, has been linked to a higher risk of ART treatment failures.^21,33,34^ Accordingly, findings from a meta-analysis of 125 studies suggest that one quarter of ALHIV experienced depression as a major adherence barrier.^23^ Apart from this, poverty was found to diminish a person's sense of self-worth, which, in the context of HIV, may also find expression in internalizing HIV-related stigma.^14^ Internalized stigma may subsequently translate into lower adherence levels.^35,36^ Adolescents might be more vulnerable to perceived stigmatization through greater observability of medication and clinic appointments by peers in school while social interactions and peer perceptions are particularly important in adolescence.^37–39^ For example, one qualitative study conducted with perinatally infected ALHIV revealed that crucial barriers to ART adherence were the fear of being seen by peers when collecting ART medication and felt uncomfortable taking medication in front of others.^37^

Building on this, our study aims at examining how the detrimental impacts of poverty on ART adherence could be mitigated. Specifically, our analysis sheds light on the multifaceted pathways through which economic empowerment may increase ART adherence among a cohort of 933 ALHIV in South Africa who were interviewed in 3 data collection waves. Our study advances existing evidence in several ways. First, we put specific focus on adolescents as a key vulnerable group, given the elevated risk of low ART adherence in this age group and the potential long-term harm associated with experiences of lifelong physical illness comorbidity and poor mental health when transitioning into adulthood. Second, our study leverages the, to date, largest cohort of ALHIV to test the associations of poverty and adherence and exploits the longitudinal structure of the data to establish the directionality of effects. Third, this is the first study, to the best of our knowledge, to use a structural equation modeling approach to elucidate the specific mechanisms underlying the relationship between economic well-being and ART adherence.

## METHODS

### Study Setting and Participants

The study took place in the Eastern Cape province of South Africa, which is the province with the highest percentage of people living below the poverty line, highest food insecurity, and the poorest basic service delivery in the country.^40,41^ Within the Buffalo City Municipality Health District, we identified a total of 81 clinics. From these, we conducted a detailed mapping of information on (1) the type of clinic (pediatric, general, antenatal care, or ARV unit), (2) the number of ALHIV estimated to receive care in each facility, (3) the number of adolescent patients on ART in each facility, and (4) the number of adolescent patients lost to follow-up, down-referred, or who had passed away. From this list, we selected 32 clinics based on the following two eligibility criteria: (1) having at least 5 ALHIV as patients and (2) being a public clinic. In addition, we included 21 smaller facilities to which ART patients in the region had been down-referred during the study baseline, thus yielding a final list of 53 clinics, community health centers, and hospitals.

In each of these health care facilities, we reviewed patient files to identify adolescents who had ever initiated ART and were aged between 10 and 19 years. Adolescents were eligible irrespective of whether they were currently on ART or lost to follow-up; those not regularly attending health facilities were traced in rural, urban, and periurban communities in collaboration with health care providers, social workers, and counselors.

**FIGURE 1.**
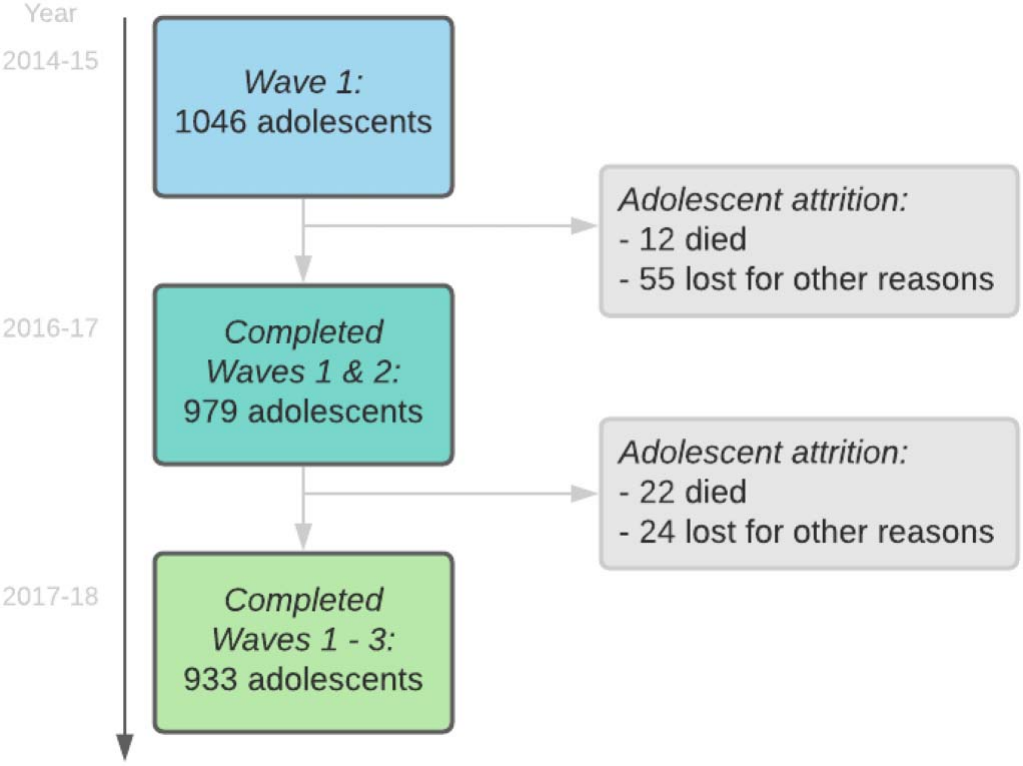
Study flow chart.

One thousand forty-six (90.2%) of study-eligible ALHIV were recruited into the study and completed baseline interviews in 2014–2015. 4.1% refused participation (either adolescent or caregiver), 0.9% had very severe cognitive delay, 3.6% were untraceable, and 1.2% no longer lived in the area. 94% (979) of the participants interviewed at baseline were reinterviewed at wave 2 of the study in 2016–2017 and 95% (933) of these at wave 3 in 2017–2018 (Fig. 1). Participants who had moved were traced across the country, including data collection in 6 of the 9 South African provinces. 34 participants could not be traced, withdrew their consent, or had passed away and were thus lost to follow-up between wave 1 and the final wave 3.

#### Ethical Procedures

The study obtained ethical approvals by the University of Cape Town (CSSR 2013/4), the University of Oxford (CUREC2/12-21), the provincial Departments of Health and Education, and participating health care facilities. If the participant was younger than 18 years old, both the adolescent and legal guardian provided written, voluntary, and informed consent to participate in each data collection wave. For participants 18 years and older, legal guardians were not involved in consent procedures. Financial incentives were not offered to ensure fully voluntary consent and avoid the potentially coercive appeal of receiving money for participation. Instead, based on consultations with an adolescent advisory group,^42^ participants received small gift packs including fruit juice and snacks, soaps, pencils, and a printed certificate of participation. If research assistants identified an urgent need for a participant to access more extensive support, for example, in case of recent rape, abuse, suicidal attempts, or severe illness, referrals were made to health and social services with the participant's–and where appropriate, caregiver's–consent.

We further took several precautions to alleviate the risk of HIV-related stigmatization for study participants. First, the research study was presented in communities in very general terms, emphasizing a focus on adolescent health and well-being. Second, to normalize participation in the study, we included a convenience sample of 456 adolescents (excluded from this analysis) who resided in the same communities and for whom a health screening suggested that their HIV status was negative. Third, for participants who were not yet aware of their HIV status at the time of the interview, research assistants were trained to use a survey version in which questions were formulated more vaguely, for instance by replacing the word “HIV” with “illness.”

### Measures

Data were collected by device-assisted face-to-face interviews that lasted between 60 and 90 minutes. Questionnaires were carefully piloted and translated from English to Xhosa and back. All questionnaires are available at http://www.youngcarers.org.za/youthpulse. The specification of all measures is detailed in Supplemental Digital Content (see Table S1, http://links.lww.com/QAI/B942).

### Outcome Variable: ART Adherence

The outcome variable was defined as ART adherence in the past 7 days. Adherence measures were drawn from the Patient Medication Adherence Questionnaire and measures developed in Botswana.^43,44^ ART adherence was defined as a dichotomous variable coded 1 (for adherence) in case self-reported adherence on weekdays and the weekend exceeded 95% and coded 0 (for non-adherence) if the respondent reported (1) having taken less than all required doses in the past 3 days, (2) having missed at least 1 dose in the past week, (3) having missed at least 1 dose on the past weekend, or (4) being currently not on ART (ie, defaulting).^45,46^ We applied a threshold of 95% based on previous classifications of ART adherence into optimal (>95%) and suboptimal (>95%) levels.^47–50^ This classification was guided by clinical evidence on variations in patients' virologic, immunologic, and clinical outcomes along different levels of ART adherence, revealing that patients with adherence levels of >95% had substantially lower rates of virologic failure, significantly less hospitalization days, and no opportunistic infections, compared with patients with adherence levels between 80% and 94.9%.^51^ The high threshold is also justified by evidence pointing to the risk of under-reporting nonadherence due to stigmatization, social desirability considerations, and recall and memory biases.^52,53^ To further alleviate potential under-reporting, we integrated questions with different framings, vignettes, and recall periods in different survey sections, thus aiming to make participants more comfortable to discuss potential nonadherence.^54^

To assess the validity of the adherence self-reports, we additionally draw on viral load measures from clinical patient files. These data were only available for a subsample of 650 ALHIV at wave 1 (ie, 70% of the full sample) and 598 adolescents at wave 2 (ie, 64% of the full sample). For patients not included in this subsample, viral load measures were either not shown in patients' clinical records or taken more than 2 years before or after the interview date. In some cases, patient files were unavailable altogether.^46,55^ Using a logistic regression, we assessed whether self-reported adherence significantly predicted patients' undetectable viral load (<50 copies/mL), controlling for factors that are likely associated with patients' adherence, including their sex, age, rural/urban location, living standards, orphanhood status, vertical/horizontal HIV infection, and perceived health status.

### Explanatory Variable: Economic Well-being

Economic well-being was captured by drawing on indicators for the top 8 most important basic necessities, selected by over 80% of the South African population in a nationally representative survey.^56^ Specifically, these included having sufficient clothes and toiletries, being able to afford 3 meals per day, having access to health care when needed, and being able to cover educational costs, including school uniforms, books, and stationary. Self-reported information on access to each of these basic necessities was aggregated into an additive continuous scale of economic well-being.

### Mediating Variables

We examined several variables potentially mediating the relationship between economic aspects and adolescents' ART adherence. Hypothesized *structural pathways of ART adherence* included self-reported information on (1) having sufficient money to travel to the clinic and (2) having sufficient food to eat when taking antiretrovirals. Hypothesized *internal pathways of ART adherence* captured psychological and emotional well-being by drawing on a combined additive scale composed of an adapted and regionally validated 14-item version of the Revised Children's Manifest Anxiety Scale^57^ and a regionally validated 10-item short version of the Child Depression Inventory.^58^ The scale was then reverse coded so that higher scores reflected lower levels of anxiety and depression and thus better mental health. Finally, we measured internalized HIV stigma, using the 6-item HIV-stigma subscale for ALHIV (ALHIV-SS), adapted and validated in this setting.^59^ The scale captured, for example, whether adolescents feel “ashamed,” “contaminated,” or “like a bad person” because of their HIV status. Again, we reverse coded the scale so that higher scores corresponded to lower levels of internalized HIV stigma and thus a more positive self-image.

### Statistical Analyses

We used a hybrid probit regression model to exploit both within-person variation as well as variation between individuals over time and thus combine the strengths of random and fixed-effects models.^60–62^ For the explanatory variable of household economic well-being, captured through access to selected basic needs, we included the individual's average value, pooled across 3 data collection waves, as well as the time-specific deviation from this average. The regression equation was thereby set up as follows:

(1) $$P\left(Adherence_{ti}=1\;|x_{it}\right)=\text{Φ}\left(\beta_{0}+\beta_{1B}\left({\bar{x}}_{i}\right)+\beta_{2W}\left(x_{it}-{\bar{x}}_{i}\right)+v_{i0}+c{'}_{it}+\varepsilon_{it}\right)$$

with $Adherence_{it}$ as the time-varying dependent variable, $\beta_{0}$ as the random intercept at the level of the individual, and $x_{it}$ as a time-varying independent variable for person *i* at time *t*.   $\beta_{1B}$ represents the person's average, that is, the between-effect of $x_{it,}\;\beta_{2W}\;\text{d}$ denotes the deviation from the average, that is,. the within-person effect of $x_{it,}\;v_{i0}$ is a random effect tied to the intercept and assumed to be normally distributed, and $\varepsilon_{it}$ represents the homoscedastic residuals. The analysis included participants' sex (time invariant), horizontal/vertical infection (time invariant), age, urban/rural residence, household size, and orphanhood (each time variant) denoted with $c{'}_{it}$ as a vector of control variables. To interpret coefficients in more intuitive terms, we additionally estimated average adjusted probabilities for the effect of household economic well-being on ART adherence.^63^ Specifically, within-person estimates refer to the association between possible reductions (or increases) in poverty and improvements (or deteriorations) in ART adherence that one individual may experience over the 3 data collection waves. By contrast, the between-person effect captures whether general differences between adolescents' poverty levels can explain variation in ART adherence rates, with both variables averaged across the 3 waves.

We additionally assessed the robustness of our findings by using alternative model specifications, namely, a pure fixed-effects and a random-effects regression model. An advantage of the former model is that estimates are unconfounded by time-invariant unmeasured factors.^64^ Yet, a drawback of the former model is that it does not accommodate for the estimation of the effect of any variable that remains constant over time (eg, sex),^65^ which the latter model can achieve.

Finally, we tested hypothesized structural and internal pathways in a structural equation model, using the average within-effect and between-effect specification from above for the explanatory and mediating variables. The structural equation allowed for simultaneously estimating the effect of household economic well-being on each hypothesized mediator as well as the effect of each mediating variable on ART adherence.^66^ All analyses were conducted in Stata 17.

## RESULTS

### Sample Characteristics

Sample characteristics are shown in Table 1. 55% (514) of participants were girls; the mean age was 13.6 years at baseline, about a third lived in rural locations, and 79% (729) had been vertically infected with HIV. Self-reported ART adherence was at 66% (615) at wave 1 and went up to 75% (700) at wave 3 (Table 1). We validated the self-reported adherence rates against viral load biomarkers in the patient subsample for which we had sufficiently detailed clinical files available. We show that self-reported adherence was associated with 1.5 times higher odds of undetectable viral load (<50 copies/mL) (Supplemental Digital Content, Table S2, http://links.lww.com/QAI/B942), thus confirming the clinical relevance of our main outcome variable. All subsequent analyses will, therefore, be based on the self-reported adherence data, which is available for the full study sample and thus not subject to possible selection biases linked to missing clinical records for some patients.

**TABLE 1.** Sample Characteristics Across Three Waves (N = 933 Adolescents)

Categorical variables	Wave 1	Wave 2	Wave 3
	n (%)	n (%)	n (%)
Continuous variables	Mean (SD)	Mean (SD)	Mean (SD)
Outcome and Predictor			
Full past week adherence	615 (66%)	605 (65%)	700 (75%)
Household poverty scale (access to selected basic needs)	6.36 (1.82)	5.54 (2.26)	5.69 (2.29)
Structural and internal mediators			
Past year lack of money to travel to the clinic			
Never	718 (77%)	828 (89%)	865 (93%)
Once or twice	120 (13%)	68 (7%)	53 (6%)
Several times	48 (5%)	19 (2%)	10 (1%)
Most of the time	47 (5%)	18 (2%)	5 (1%)
Had shortages of food to eat with medication	379 (43%)	432 (48%)	275 (31%)
Internalized stigma scale	1.94 (1.67)	1.80 (1.74)	0.26 (0.82)
Anxiety and depression score	3.40 (3.88)	1.64 (2.84)	1.33 (2.42)
Sociodemographic information			
Vertically infected with HIV (time invariant)	729 (79%)		
Female (time invariant)	514 (55%)		
Age	13.56 (2.88)	15.07 (2.88)	16.26 (2.90)
Rural location	249 (27%)	230 (25%)	223 (24%)
Household size	6.77 (2.90)	6.18 (3.82)	5.73 (2.96)
Orphan hood (lost mother, father, or both)	545 (58%)	560 (60%)	640 (69%)

Notes: N = 933 adolescents.

Adolescents reported that their households had on average access to 6 of 8 basic necessities across all 3 waves. The within-person changes in household poverty over time are summarized in Supplemental Digital Content (see Table S3, http://links.lww.com/QAI/B942). Namely, 14.33% of adolescents reported having experienced a decrease in poverty equivalent to owning one additional basic necessity between the first and second data collection wave, and 15.80% of adolescents reported the same reduction in their poverty level between the second and third wave. Similarly, 9.74% of participants between the first and second data collection wave and 20.83% of participants between the second and third wave experienced more substantial reductions in poverty, equivalent of securing access to at least 3 additional basic necessities. The person-level intraclass correlation in economic well-being across the 3 time points was at 0.081 [95% confidence interval (CI): 0.04 to 0.12].

Seventy-seven percent (718) of adolescents living with HIV reported having sufficient financial resources to travel to the clinic in wave 1, compared with a considerably higher rate of 93% (865) in wave 3. The rate of adolescents who indicated that shortages of food hampered their ART adherence ranged between 48% (432) at wave 2% and 31% (275) at wave 3. Self-reported symptoms of internalized stigma, as well as depression and anxiety, varied more considerably between time points.

### Household Economic Well-being and ART Adherence

Table 2 below shows the hybrid probit regression results estimating the association between household economic well-being and adolescents' ART adherence. Our regression results suggest that within-person improvements in economic well-being correspond to statistically significant increases in ART adherence among adolescents living with HIV.

**TABLE 2.** Multivariable Probit Regression: Association Between Household Economic Well-being (Access to Basic Needs) and ART Adherence

	Coefficient (SE)	Lower 95% CI	Upper 95% CI	*P*
Explanatory variable				
Economic well-being— *between*	0.06* (0.02)	0.01	0.11	0.013
Economic wellbeing— *within*	0.04** (0.02)	0.01	0.07	0.013
Control variables				
Female—*between*	−0.02 (0.06)	−0.14	0.09	0.694
Horizontally infected—*between*	−0.28** (0.09)	−0.45	−0.12	0.001
Age—*between*	−0.01 (0.01)	−0.03	0.02	0.569
Age—*within*	−0.13*(0.06)	−0.25	−0.01	0.031
Rural residence—*between*	0.04 (0.07)	−0.10	0.19	0.544
Rural residence—*within*	0.09 (0.16)	−0.23	0.42	0.576
Household size—*between*	−0.00 (0.01)	−0.02	0.02	0.825
Household size—*within*	0.01 (0.01)	−0.01	0.04	0.199
Orphanhood—*between*	0.05 (0.07)	−0.08	0.19	0.436
Orphanhood—*within*	−0.10 (0.13)	−0.35	0.16	0.467
Time point (follow-up)	0.35*** (0.08)	0.18	0.51	<0.001

N = 933 adolescents, 2799 observations over 3 waves.

*P < 0.05, **P < 0.01, ***P < 0.001.

Household well-being is an additive index composed of access to and ownership of 8 selected basic needs.

**FIGURE 2.**
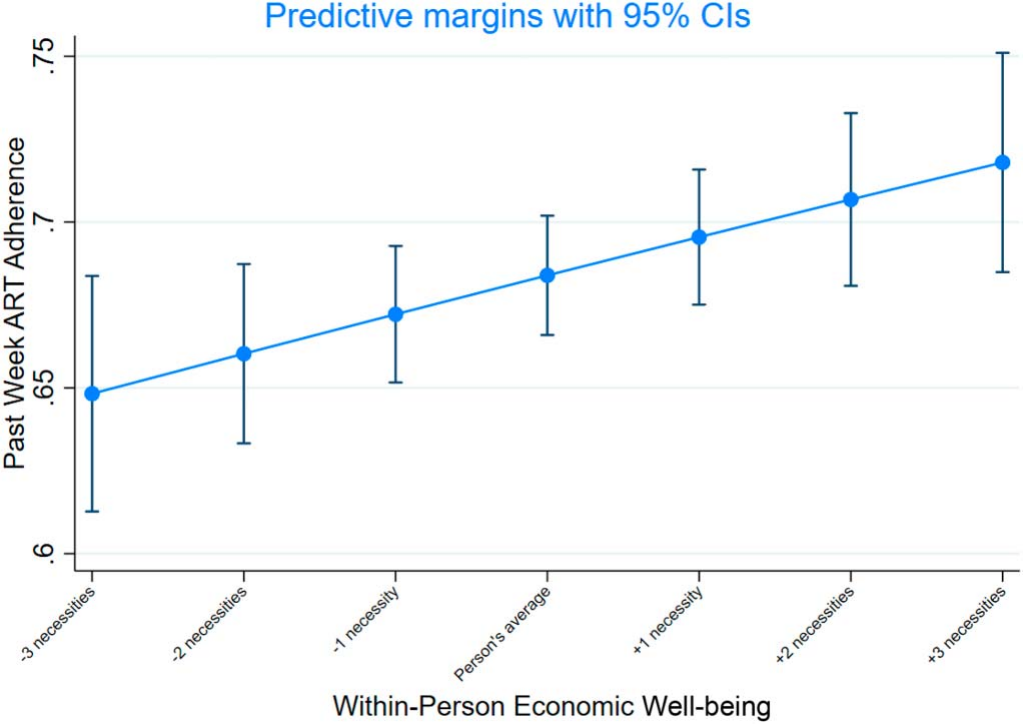
Adjusted average predicted probabilities of ART adherence at different levels of the explanatory variable: within-person estimates. Notes: Average-adjusted probabilities are reported as average marginal effects, adjusted for sex, vertical/horizontal infection, age, rural/urban location, household size, and orphanhood status. The x-axis displays decreases and increases in economic well-being from the individual's own average.

**FIGURE 3.**
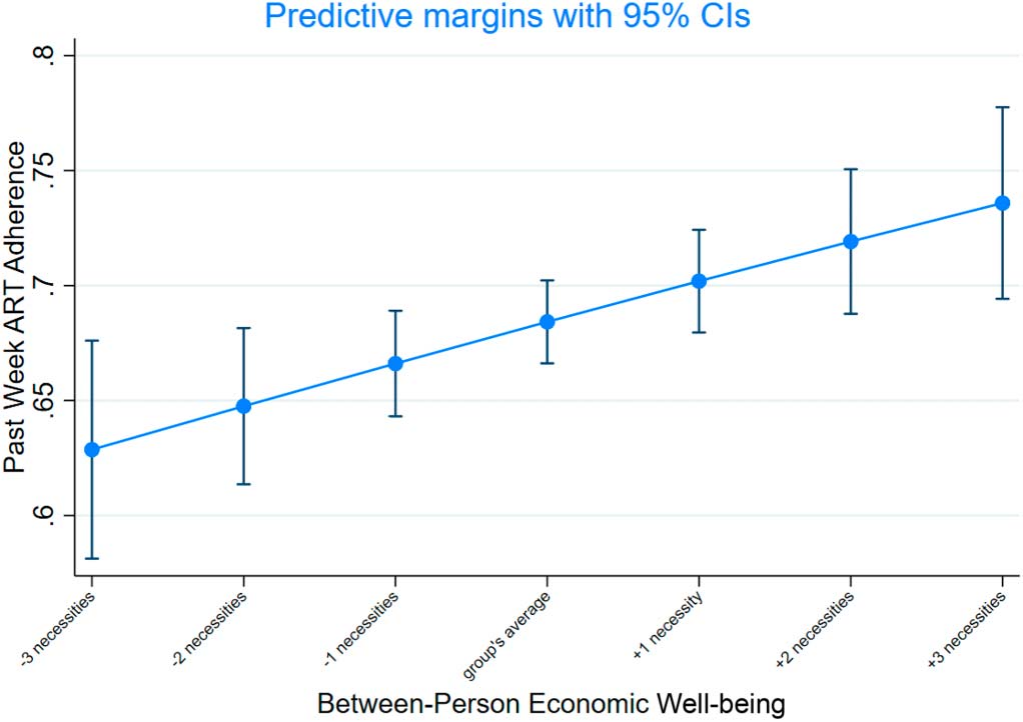
Adjusted average predicted probabilities of ART adherence at different levels of the explanatory variable: Between-person estimates. Notes: Average adjusted probabilities are reported as average marginal effects, adjusted for sex, vertical/horizontal infection, age, rural/urban location, household size, and orphanhood status. The x-axis displays decreases and increases from the group's average level in economic wellbeing, pooled across 3 waves.

Similarly, between-person effects reveal that adolescents who experienced on average higher levels of household economic well-being across all 3 waves, captured through access to selected basic needs, had higher levels of ART adherence. To present the regression results in more intuitive terms, we predicted the average adjusted probability of ART adherence for different levels of household economic wellbeing *within* (Fig. 2) and *between* (Fig. 3) individuals. Notably, as displayed in Figure 2, having access to one additional basic necessity above the average level reported by the individual was associated with a 2 percentage point higher probability of this adolescent's past week ART adherence (*P* = 0.013). Having access to 3 additional basic necessities above the individual's average would yield a 4 percentage point higher likelihood of past week ART adherence.

Between adolescents, the likelihood of ART adherence was 2 (6) percentage points higher (*P* = 0.013) for those who had on average one (3) additional basic necessity, pooled across waves (Fig. 3).

Finally, sensitivity analyses based on separate fixed-effects and random-effects regression models reproduce the same pattern of findings, corroborating the positive and significant link between higher economic well-being and higher ART adherence (Supplemental Digital Content, Tables S4–S5, http://links.lww.com/QAI/B942).

### Structural and Internal Pathways to Improved ART Adherence

**FIGURE 4.**
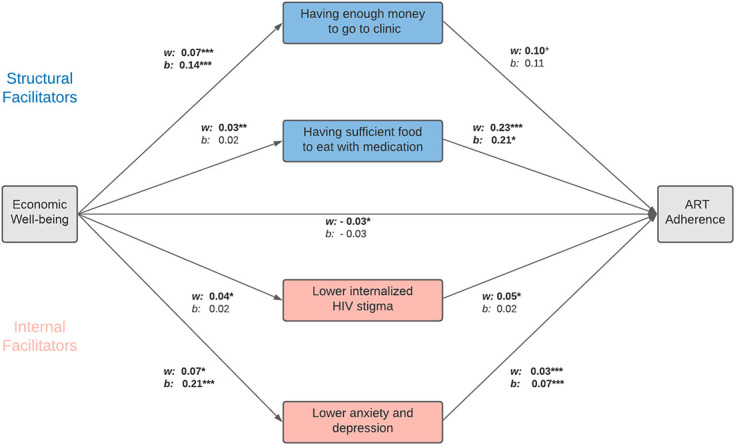
Hybrid model in a structural equation model framework: structural and internal economic pathways to improved art adherence. Notes: *P < 0.05, **P < 0.01, ***P < 0.001, significant associations in bold. N = 2785 due to item missings and list wise deletion. w denotes within-person effects and b denotes between-person effects. Pathways with binary dependent variables were estimated in a probit regression model, and pathways with continuous dependent variables were estimated in a linear regression model. Analyses control for participants' sex (time invariant), age, household size, and urban/rural location; standard errors are robust and clustered at the individual level. Model based on nonadaptive Gauss–Hermite quadrature to facilitate convergence.

Figure 4 shows the hypothesized structural and internal economic pathways to improved ART adherence in a structural equation path model, whereby *w* denotes the within-person and *b* the between-person estimates for each association (for detailed results see Supplemental Digital Content, Table S6, http://links.lww.com/QAI/B942). Regarding structural pathways, we found that within-person and between-person increases in access to basic needs increased the likelihood that adolescents' had sufficient funds to travel to the clinic [$\beta_{W}$ = 0.07, 95% CI: (0.04 to 0.11), *P* < 0.001, $\beta_{B}$ = 0.14, 95% CI: (0.10 to 0.19), *P* < 0.001], which subsequently translated into a tentatively higher probability of ART adherence [$\beta_{W}$ = 0.10, 95% CI: (0.00 to 0.21), *P* = 0.05, $\beta_{B}$ = 0.11, 95% CI (−0.04 to 0.25), *P* = 0.14] Furthermore, within-person improvements in economic well-being (not between-person improvements) were associated with the ability to afford sufficient food to eat with ART medication [$\beta_{W}$ = 0.03, 95% CI: (0.00 to 0.06), *P* = 0.02, $\beta_{B}$ = 0.02, 95% CI (−0.02 to 0.06), *P* = 0.39] Having sufficient food was associated with increases in the likelihood of ART adherence, both within and between individuals [$\beta_{W}$ = 0.23, 95% CI: (0.10 to 0.37), *P* < 0.001,$\beta_{B}$ =0.21, 95% CI: (0.04 to 0.37), *P* = 0.02].

Turning to internal pathways, adolescents who experienced poverty reductions over time reported lower levels of internalized HIV stigma, whereas average differences between adolescents in economic well-being were not significantly associated with internalized HIV stigma [$\beta_{W}$ = 0.04, 95% CI: (0.00 to 0.07), *P* = 0.03 $,\;\beta_{B}$ = 0.02, 95% CI: (−0.02 to 0.06), *P* = 0.40]. In addition, a within-person improvement regarding internalized HIV stigma led to a higher probability of ART adherence [$\beta_{W}$ = 0.05, 95% CI: (0.00 to 0.09), *P* = 0.04,$\beta_{B}$ = 0.02, 95% CI: (−0.05 to 0.08), *P* = 0.63]. Finally, increases in household economic well-being were associated with improved mental health status and thus a reduced likelihood of anxiety and depression symptoms, both for changes within individuals over time as well as for differences in the between-person average across all 3 waves [$\beta_{W}$ = 0.07, 95% CI: [0.01 to 0.14], *P* = 0.03 $,\;\beta_{B}$ = 0.21, 95% CI (0.04 to 0.37), *P* = 0.02] Improved within-person and between-person mental health status, in turn, was associated with a higher probability of ART adherence [$\beta_{W}$ = 0.03, 95% CI: (0.01 to 0.06), *P* = 0.004,$\beta_{B}$ = 0.07, 95% CI: (0.04 to 0.10), *P* < 0.001].

## DISCUSSION

This study revealed critically low levels of ART adherence in a cohort of ALHIV in South Africa, far below national targets, similar to nationally representative data.^5^ Understanding and alleviating the factors that hamper ART adherence is essential for reaching the UNAIDS HIV treatment target. We contribute to this understanding by revealing important links between economic strengthening and adolescents' ART adherence. Specifically, we find that economic well-being may improve ART adherence through both internal and structural pathways. Having sufficient financial means to afford transport to the clinic and having sufficient food when taking ARVs significantly increased the likelihood of past week ART adherence among adolescents. Although living in poverty can have detrimental psychological impacts, we found that adolescents with better mental health functioning and lower internalized HIV stigma were more likely to take their medication regularly. Our findings thus corroborate those from previous, largely qualitative, studies that have highlighted the importance of financial resources for food, transport to the clinic, HIV-related stigma, and mental health for adolescents' ART adherence.^18,19,26,37^ To the best of our knowledge, we present the first analysis that explicitly models these respective pathways by applying a structural equation model.

A recent meta-analysis of 10 interventions which aimed to increase medication adherence in adolescents living with ART reported an insignificant pooled effect size close to zero, thus suggesting that more evidence on effective strategies to boost adolescents' adherence rates is urgently needed.^67^ Two of the 10 included studies were conducted in South Africa, whereby one assessed a family-based psychosocial intervention and found no significant increases in adolescents' ART adherence.^68^ The other study assessed the impact of social protection in the form of “cash + care” on adolescents' ART adherence–including, among others, governmental cash transfers, school feeding, HIV support groups, and positive parental supervision–finding that without any “cash + care” support, nonadherence was at 54% but could be reduced to 39%–41% with additional social protection.^69^ Our model predicts that adolescents who receive one additional basic necessity can increase their predicted probability of ART adherence from a baseline probability of 66% by 2 percentage points. Although this effect remains relatively small in magnitude, it is still highly relevant in view of the null effects of other studies, thus generating important policy implications. In settings characterized by high levels of poverty and deprivation, economic empowerment approaches targeted at adolescents and their families can help to directly alleviate the structural barriers of ART adherence. Approaches could include unconditional cash transfers as well as focused “cash in kind” interventions such as providing transport subsidies for reaching the clinic or distributing food parcels and vouchers.^6^ Economic empowerment programs may also have the potential to indirectly improve mental health functioning^14,29,70^ and thus also help alleviate internal barriers of ART adherence. Future research should specifically test the impact of economic strengthening programs on the financial as well as psychological well-being of beneficiaries, assess the suitability of such programs for young people specifically, and examine whether interventions are more effective if targeted at the family as a whole or at adolescents alone.^32^

In addition, we observed that changes in a person's economic well-being between study waves had a significant impact on their ART adherence. This highlights the importance of programs that help individuals and households to smooth consumption over time and to more effectively cope with possible income shocks. Interventions to promote saving and financial inclusion as well as insurance programs that are accessible for those with low incomes could help to build resilience against income shocks.^32,71^ An example is the multicomponent *Suubi-Maka* intervention, consisting of mentoring, financial literacy training, and access to savings accounts, and implemented and tested with AIDS-affected youth in Uganda.^72,73^ However, although the program led to significant improvements in ART adherence, self-reported health status, and significant reductions in HIV-related internalized and enacted stigma,^72,73^ there may need to be adaptations for a cost-efficient and sustainable regional scale-up.

Our analyses are subject to several limitations. First, we cannot make causal claims due to possible confounding through time-variant factors that were not measured (such as potential peer influences and various experiences at school, at home, or at the clinic). Second, some of the examined pathways might be characterized by reverse causality. For example, several studies have found tentative evidence suggesting that antiretroviral drugs' side effects may increase the risk of experiencing depressive symptoms among some patients living with HIV,^74,75^ whereas others have rejected this hypothesis.^76^ It is also possible that poor ART adherence–and associated deterioration of patients' health–increases internalized HIV stigma.^77^ Third, we relied on adolescents' self-report for all included measures. It is possible that adolescents were hesitant to disclose having missed any doses in the past week due to social desirability considerations, fear of possible stigmatization, or simply misremembering. However, we alleviate this concern both by validating self-reported adherence levels with clinical viral load data and by using a short recall period of 1 week for the outcome measure. Fourth, some study participants could not be traced in the second and third survey wave. Although the level of attrition was very low in this cohort study, it is possible that those lost to follow-up include the most vulnerable and marginalized ALHIV, which would imply that the estimated adherence rate is biased upward. Fifth, for 3 of 4 pathways to ART adherence, associations were not consistently significant across within-person and between-person estimates. In consequence, we are most confident about the importance of mental health–related pathways to improved ART adherence, considering that the mediation effect was statistically significant both across adolescents and within individuals. Finally, our structural equation model only included internalized HIV stigma, thus possibly neglecting other dimensions of HIV-related stigma such as experiences of discrimination or social exclusion.^78,79^ These other forms of stigma can erode a person's social capital and community safety nets, which, in turn, could translate into lower ART adherence through the lack of positive peer reinforcement.^80^

Despite these limitations, the study presents important and novel longitudinal evidence from the largest cohort of ALHIV in South Africa. Most importantly, our findings underline the importance of integrating social protection mechanisms, to address the unique social, economic, and spatial circumstances of adolescents and to address essential needs, risks, and vulnerability. Existing literature has failed to adequately describe that generalized HIV risk is underpinned by socioeconomic and spatial positions of disadvantage that intersect and compound. Better aligning the social protection agenda with the new UNAIDS Global AIDS Strategy 2021–2026 and harnessing the contribution of social protection to health outcomes, namely, by cash and food transfers, is essential to ending AIDS as a public health threat by 2030. The strategy aims at building *“[s]ystems for health and social protection schemes that support wellness, livelihood, and enabling environments for people living with, at risk of, or affected by HIV.”*^81^ Understanding the specific treatment challenges and needs of young people living in poverty is essential for ensuring that the design of such social protection schemes is youth friendly.
